# Effects of Information-Motivation-Behavioural Skills Model-Based Exercise and Mindfulness-Based Stress Reduction on Chronic Obstructive Pulmonary Disease With Anxiety and Depression

**DOI:** 10.62641/aep.2281

**Published:** 2026-10-15

**Authors:** Wenrong Liu, Zhihuan Mi, Fen Su

**Affiliations:** ^1^Surgical Outpatient Clinic, The Second Affiliated Hospital of Dalian Medical University, 116021 Dalian, Liaoning, China; ^2^Pediatric and Adolescent Ward, Kailuan Mental Health Center, 063000 Tangshan, Hebei, China; ^3^Outpatient Department, The First Affiliated Hospital of University of Science and Technology of China, Division of Life Sciences and Medicine, University of Science and Technology of China, 230001 Hefei, Anhui, China

**Keywords:** chronic obstructive pulmonary disease, anxiety, depression, exercise rehabilitation, mindfulness-based stress reduction, information motivation behavioral skills model

## Abstract

**Background:**

This study aims to evaluate the effects of an information motivation behavioral skills (IMB) model-based exercise combined with mindfulness-based stress reduction (MBSR) on patients with chronic obstructive pulmonary disease (COPD) and comorbid anxiety and depression.

**Methods:**

This retrospective study included 168 patients with COPD and comorbid anxiety and depression who visited the Department of Respiratory Medicine of The Second Affiliated Hospital of Dalian Medical University between January 2023 and December 2024. Patients were divided into a routine care group (n = 84) and a combined treatment group (n = 84, receiving 12-week IMB model-based exercise rehabilitation combined with MBSR) based on the care they received. Changes in the Hamilton Anxiety Scale (HAMA), Hamilton Depression Scale (HAMD), modified Medical Research Council (mMRC) dyspnea scale, 6-minute walk distance (6MWD) and pulmonary function indicators were compared between the two groups before and after the treatment.

**Results:**

After treatment, the combined treatment group showed significantly greater improvements than the routine care group in reducing HAMA, HAMD, increasing 6MWD and improving forced vital capacity (FVC) and forced expiratory volume in one second (FEV1) (all *p* < 0.001). The distribution of mMRC grades also differed significantly between the two groups post intervention (χ^2^ = 10.630, df = 3, *p* = 0.013), favoring the combined treatment.

**Conclusions:**

The IMB model-based exercise rehabilitation and MBSR was associated with reduced the anxiety and depressive symptoms, improved dyspnea, enhanced exercise tolerance and better lung function in patients with COPD and comorbid anxiety and depression. These findings suggest that this integrated mind–body rehabilitation may provide a reference for comprehensive COPD management.

## Introduction

Chronic obstructive pulmonary disease (COPD) is a common respiratory disease with substantial morbidity and mortality [1]. In addition to respiratory symptoms, additional common symptoms of patients with COPD include fatigue and difficulties in managing their physical and psychosocial needs [2]. A longitudinal study reported that patients with COPD had nearly twice the risk of developing depression compared with matched non COPD controls, with incidence rates of 11.4 and 5.7 per 1000 person years, respectively [3]. Anxiety and depression can interfere with the treatment of COPD, creating a vicious cycle of declining health status and increasing psychological burden [4]. However, current clinical management remains largely focused on pharmacotherapy and conventional rehabilitation, with insufficient attention paid to psychological comorbidities. Traditional single-approach models fail to meet the dual physiological and psychological rehabilitation needs of patients with COPD.

In traditional COPD management, exercise rehabilitation is widely recognized as an effective method. It can substantially improve patients’ exercise tolerance, dyspnea symptoms, and health related quality of life, and is associated with reduced rehospitalization rates and improved survival [5]. However, traditional exercise programs focus on physical function recovery, and aerobic exercise alone may have limited effects on depression [6]. Mindfulness-based stress reduction (MBSR) aims to cultivate inner balance and spiritual growth through non-judgmental, non-reactive, and present-moment awareness of psychological states and experiences. These practices are believed to reduce suffering through interconnected processes, promote a present-centred awareness model, and enable individuals to relate to experiences with acceptance and compassion rather than avoidance, control, or suppression [7]. However, MBSR alone is unlikely to directly improve the physical functions of patients with COPD and cannot break the vicious cycle of declining health status and increasing psychological burden.

The information motivation behavioral skills (IMB) model identifies three core potential factors of healthy behaviours: information, motivation, and behavioural skills. Information refers to accurate knowledge about a specific behaviour. Motivation encompasses both personal and social components. Individual motivation reflects an individual’s attitudes or beliefs, whereas social motivation reflects perception of social support. Behavioural skills refer to a person’s self-efficacy and ability to perform an action effectively [8]. Treatments designed based on the IMB model can systematically address cognitive, motivational, and skill barriers in the process of patients’ transformation from “knowing” to “doing” laying the foundation for long-term maintenance of healthy behaviours.

Based on the above background, this study aims to explore the association of an IMB model-based exercise combined with MBSR, with outcomes in patients with COPD and comorbid anxiety and depression.The findings may provide evidence for breaking the vicious cycle of the disease management and improving the long-term prognosis of patients.

## Materials and Methods

### General Information

This retrospective study extracted data of patients with COPD and comorbid anxiety and depression from the electronic medical record system of the Department of Respiratory Medicine of The Second Affiliated Hospital of Dalian Medical University between January 2023 and December 2024. All eligible patients were informed about the availability of the 12-week COPD Mind–Body Integration Rehabilitation Program. Participation in this program was voluntary and based on patient preference after consultation with their treating physician. Patients who chose to participate and completed the program were included in the combined treatment group. Those who declined the program and only received standard outpatient follow-up and basic health education were included in the routine care group. No active program was assigned by the researchers; instead, patients were divided into two groups based on whether they had received the 12-week combined program, as documented in their medical records.

Inclusion criteria: (1) meeting the diagnostic criteria of stable COPD according to the Global Initiative for Chronic Obstructive Lung Disease (GOLD), with moderate-to-severe disease severity (GOLD stages II–III) [9]; (2) aged 40–75 years; (3) Hamilton Anxiety Scale (HAMA) scores ≥14 and Hamilton Depression Scale (HAMD-17) scores ≥17 [10, 11]; and (4) complete clinical data.

Exclusion criteria: (1) presence of severe heart, liver, kidney or other major organ failure; (2) history of mental illness or cognitive impairment that could interfere with cooperation with scale assessment; (3) contraindications to exercise, such as severe osteoporosis or joint deformity; (4) participation in other similar rehabilitation or psychotherapy programs within the past 3 months; or (5) poor adherence.

The research followed the guiding principles of the Declaration of Helsinki. Written informed consent was obtained from all participants. This study was approved by the Ethics Committee of The Second Affiliated Hospital of Dalian Medical University (Ethics Approval No.: KY2025-310-01-01).

All assessors completed standardized training in administering HAMA and HAMD before data collection to ensure consistency. The Fifth Edition of Diagnostic and Statistical Manual of Mental Disorders (DSM-5) diagnoses of anxiety and depressive disorders were established by two qualified psychiatrists based on medical record review and clinical interview notes, with any disagreements resolved by consensus. None of the participants received any psychotropic medications or formal psychotherapy during the study period. Baseline psychiatric symptom severity was documented and included as a covariate in the statistical analysis to control for potential confounding effects.

A total of 212 consecutive patients with COPD and comorbid anxiety and depression were assessed for eligibility between January 2023 and December 2024. Among them, 6 were excluded due to incomplete clinical data, 14 due to other respiratory diseases, 18 due to contraindications to exercise, and 6 because they had participated in similar rehabilitation programs in the past 3 months. Finally, 168 eligible patients were included and divided into a routine care group (n = 84) and a combined treatment group (n = 84) based on the type of care documented in their medical records.

### Treatment Methods

#### Routine Care Group

Routine care consisted of: (1) patient education: basic information regarding COPD etiology, symptoms, treatment plans, and medication precautions was provided through verbal explanations and brochures; (2) basic rehabilitation guidance: patients were instructed to perform abdominal breathing and pursed-lip breathing twice daily for 15–20 minutes per session; (3) routine follow-up: weekly follow-ups by phone calls were conducted to monitor symptom changes and medication adherence; and (4) lifestyle recommendations: patients were advised to quit smoking, limit alcohol consumption, maintain a balanced diet, avoid respiratory infections, and engage in appropriate home-based exercise.

#### Combined Treatment Group

In addition to routine care, the combined treatment group received a 12-week IMB model-based exercise rehabilitation combined with MBSR. The program was jointly implemented by a multidisciplinary team consisting of respiratory physicians, rehabilitation therapists, psychotherapists and specialist nurses. Specifically, it was designed based on the three core components of the IMB model:

(1) Information acquisition stage (weeks 1–2): Through one-on-one interviews, special lectures, instructional videos covering COPD-related knowledge, the importance and specific methods of exercise rehabilitation, and the core principles and practice techniques of MBSR. Special lectures (60 minutes) were conducted, and customized treatment manuals were distributed.

(2) Motivation enhancement stage (weeks 3–4): One-on-one counselling sessions were conducted to understand patients’ rehabilitation needs, psychological concerns and physical discomfort. Rehabilitation confidence was enhanced through sharing successful rehabilitation cases and organizing patient group exchanges. Personalized motivational strategies were used to provide timely affirmation and encouragement for patients’ progress in practice.

(3) Behavioural skills training stage (weeks 5–12): Exercise rehabilitation and MBSR training were implemented concurrently.

(a) Exercise rehabilitation training [12]: Based on the results of the 6-minute walk test, personalized exercise prescriptions were developed. The program included walking training (initial intensity of 50%–60% of the maximum walking speed, 20 minutes each time, three times a week, gradually increased to 30–40 minutes), respiratory muscle training (using a breathing training device, 15 minutes each time, twice daily), and limb strength training (e.g., dumbbell curls and wall squats, 20 minutes each time, twice weekly).

(b) MBSR training [13]: A standardized curriculum was adopted, including mindful breathing, body scan, mindful walking and other exercises. An offline group training session was conducted weekly (90 minutes), and 15–20 minutes of home practice tasks were assigned daily. The instructor provided online comments and feedback.

### Observation Indicators

All indicators were extracted by assessors who were not involved in the delivery of the rehabilitation program. The assessments were performed within one week before the start of the program and one week after the end of the treatment.

(1) Psychological state assessment:

(a) Anxiety symptoms: The HAMA scale consists of 14 items, each scored 0–4, with a total score range of 0–56. A total score of ≥14 points indicates clinically significant anxiety, and higher scores reflect more severe anxiety symptoms [10];

(b) Depressive symptoms: The HAMD-17 scale consists of 17 items. Nine items (depressed mood, guilt, suicide, work and loss of interest, retardation, psychic anxiety, somatic anxiety, hypochondriasis) are rated from 0 to 4, and eight items (insomnia initial, insomnia middle, insomnia delayed, agitation, gastrointestinal somatic symptoms, general somatic symptoms, genital symptoms, loss of insight, loss of weight) are rated from 0 to 2. The total score ranges from 0 to 52. Total scores are interpreted as follows: 0–7 = normal, 8–16 = mild depression, 17–23 = moderate depression, and ≥24 = severe depression [11, 14].

(2) Dyspnoea severity: The modified Medical Research Council (mMRC) dyspnea scale was used to assess dyspnoea during daily activities. Dyspnoea was graded on a scale from 0 (dyspnoea only during strenuous exercise) to 4 (unable to leave the house due to severe dyspnoea or dyspnoea when dressing). Higher scores indicate more severe dyspnoea. The mMRC scale is easy to use in patients with COPD and correlates well with quality of life and prognosis [15].

(3) Exercise endurance: The 6-minute walk distance (6MWD) test was used to evaluate patients’ exercise tolerance and functional status. The test was performed on a flat 30-meter corridor according to standardized guidelines, and the farthest distance walked in 6 minutes was recorded. This test is a core objective indicator of functional status for patients with COPD [16].

(4) Lung function: Forced vital capacity (FVC) and forced expiratory volume in one second (FEV1) are reported in liters. These are objective physiological indicators for diagnosing and assessing the severity of airflow limitation.

### Feasibility and Safety Indicators

Feasibility analyses included the full cohort of 84 patients who initiated the combined treatment program, following a modified intention-to-treat (mITT) principle that incorporates all participants with at least one post treatment assessment. Four patients (4.8%) discontinued the full 12 week program prematurely due to disease exacerbation (n = 2) or personal schedule conflicts (n = 2). These patients underwent their final outcome assessments at the time of discontinuation and their data were included in the analyses. Therefore, the protocol completion rate for the full 12 week intervention was 95.2% (80/84). Among the 80 patients who completed the entire protocol, home practice adherence for MBSR (≥4 days per week) was 87.5% (70/80). No treatment-related adverse events were reported.

### Statistical Analysis

SPSS 21.0 software (IBM Corp., Armonk, NY, USA) was used for statistical analysis. Normality of continuous variables was assessed using the Shapiro–Wilk test; all data were approximately normally distributed. For continuous variables (HAMA, HAMD, 6MWD, FVC, FEV1), paired *t*-tests were used for within-group comparisons and independent-samples *t*-tests for between-group comparisons. For the ordinal mMRC scores, descriptive statistics are presented as frequency and percentage [n (%)] across severity levels. Between‑group differences in the distribution of mMRC grades at each time point were compared using the Pearson chi‑square test (or Fisher’s exact test when expected frequencies <5). Within group comparisons (pre: post treatment) were performed using the Wilcoxon signed rank test. Categorical data were expressed as percentages (%), and chi-square (χ^2^ tests were used for comparisons between groups. To evaluate the effects of time, group, and their interaction, a 2 (time: pre-treatment vs. post-treatment) × 2 (group: routine care vs. combined treatment) repeated-measures analysis of variance (ANOVA) was conducted for outcome measures. Partial eta squared ($\eta^{2}$) was reported as a measure of effect size. All reported *p* values are two-tailed, and a *p* < 0.05 was considered statistically significant.

## Results

### Comparison of Baseline Data Between the Two Groups

There were no statistically significant differences in baseline data including age, sex, BMI, disease duration, smoking history, GOLD classification, and the number of comorbidities between the two groups of patients (*p* > 0.05), indicating comparability (Table 1).

**Table 1.** **Comparison of baseline data between the two groups**.

Feature		Routine care group (n = 84)	Combined treatment group (n = 84)	*t*/χ^2^	*p*
Age (years, $\bar{x}$ ± s)		59.81 ± 4.65	59.62 ± 4.77	0.258	0.797
Gender (n, %)				0.026	0.873
	Male	54 (64.29)	53 (63.10)		
	Female	30 (35.71)	31 (36.90)		
BMI (kg/m^2^, $\bar{x}$ ± s)		22.81 ± 2.75	22.35 ± 2.69	1.095	0.275
Disease duration (years, $\bar{x}$ ± s)		4.01 ± 1.08	4.02 ± 1.36	–0.069	0.945
Smoking history (n, %)				0.105	0.746
	Yes	54 (64.29)	56 (66.67)		
	No	30 (35.71)	28 (33.33)		
GOLD classification (n, %)				0.024	0.876
	Level 2	48 (57.14)	47 (55.95)		
	Level 3	36 (42.86)	37 (44.05)		
Number of comorbidities (n, %)				0.139	0.933
	0	18 (21.43)	20 (23.81)		
	1	42 (50.00)	41 (48.81)		
	2	24 (28.57)	23 (27.38)		

Note: BMI, body mass index; GOLD, Global Initiative for Chronic Obstructive Lung Disease. Comorbidities included hypertension and diabetes; the number of comorbidities was categorized as 0, 1, 2.

### Comparison of Psychological State Indicators Between the Two Groups Before and After Treatment

Before the treatment, there were no significant differences in HAMA and HAMD scores between the two groups (*p* > 0.05). After the treatment, both groups showed significant reductions in HAMA and HAMD scores compared with baseline levels. The combined treatment group exhibited significantly lower post-treatment HAMA and HAMD scores than the routine care group (*p* < 0.001). See Table 2 for details.

**Table 2.** **Comparison of psychological state indicators before and after treatment in the two groups**.

Group	HAMA score ($\bar{x}$ ± s)		HAMD score ($\bar{x}$ ± s)	
	Before treatment	After treatment	Before treatment	After treatment
Routine care group (n = 84)	19.87 ± 0.34	9.57 ± 0.65***	13.14 ± 1.35	5.69 ± 0.93***
Combined treatment group (n = 84)	19.88 ± 0.36	8.37 ± 0.67***	13.19 ± 1.14	4.62 ± 0.98***
*t*	–0.220	11.821	–0.247	7.262
*p*	0.826	<0.001	0.805	<0.001

Note: HAMA, Hamilton Anxiety Scale; HAMD, Hamilton Depression Scale. ***p < 0.001 compared with baseline within the same group (paired *t*-test).

### Comparison of Dyspnoea Severity and Exercise Tolerance Between the Two Groups Before and After Treatment

Before the treatment, there were no significant differences in mMRC scores and 6MWD between the two groups (*p* > 0.05). After the treatment, the distribution of mMRC scores differed significantly between the two groups (χ^2^ = 10.630, *p* = 0.013), with the combined treatment group showing a higher proportion of patients with lower mMRC scores. 6MWD increased significantly in both groups, with the combined treatment group showing significantly longer 6MWD than the routine care group (*p* < 0.001). See Tables 3 and 4 for details.

**Table 3.** **Comparison of mMRC scores between the two groups before and after treatment**.

Group	Before treatment			After treatment			
	2	3	4	1	2	3	4
Routine care group (n = 84)	5 (5.95)	60 (71.43)	19 (22.62)	20 (23.81)	50 (59.52)	12 (14.29)	2 (2.38)
Combined treatment group (n = 84)	4 (4.76)	62 (73.81)	18 (21.43)	40 (47.62)	35 (41.67)	7 (8.33)	2 (2.38)
χ ^2^			0.171				10.630
*p*			0.918				0.013

Note: mMRC, modified Medical Research Council dyspnea scale; data are presented as frequency (percentage) across severity levels. χ^2^, Chi-square test statistic for between-group comparisons. Within-group comparisons (pre-: post-treatment) were performed using the Wilcoxon signed-rank test: routine care group Z = –7.108, *p* < 0.001; combined treatment group Z = –7.622, *p* < 0.001.

**Table 4.** **Comparison of 6MWD scores between the two groups before and after treatment**.

Group	6MWD (meters, $\bar{x}$ ± s )	
	Before treatment	After treatment
Routine care group (n = 84)	162.28 ± 12.35	289.16 ± 15.32***
Combined treatment group (n = 84)	163.89 ± 11.58	336.87 ± 20.18***
*t*	–0.871	–17.257
*p*	0.385	<0.001

Note: 6MWD, 6 minute walk distance; *t*, independent samples *t*-test statistic; ***p < 0.001 compared with baseline within the same group (paired *t*-test for 6MWD; Wilcoxon signed rank test for mMRC).

### Comparison of Pulmonary Function Indicators Before and After Treatment Between the Two Groups

Before the treatment, there were no significant differences in FVC and FEV1 between the two groups (*p* > 0.05). After the treatment, FVC and FEV1 in both groups increased compared with baseline, and the combined treatment group showed significantly higher levels than the routine care group (*p* < 0.001). See Table 5 for details.

**Table 5.** **Comparison of pulmonary function indicators before and after treatment in the two groups**.

Group	FVC (liters, $\bar{x}$ ± s)		FEV1 (liters, $\bar{x}$ ± s)	
	Before treatment	After treatment	Before treatment	After treatment
Routine care group (n = 84)	2.19 ± 0.22	2.47 ± 0.29***	1.37 ± 0.10	1.52 ± 0.17***
Combined treatment group (n = 84)	2.16 ± 0.23	2.77 ± 0.31***	1.35 ± 0.10	1.72 ± 0.19***
*t*	1.050	–6.465	1.206	–7.083
*p*	0.295	<0.001	0.230	<0.001

Note: FVC, forced vital capacity; FEV1, forced expiratory volume in one second; ***p < 0.001 compared with baseline within the same group (paired *t*-test).

### Repeated-Measures ANOVA for All Outcomes

Table 6 summarizes the repeated-measures ANOVA results. The time × group interaction was significant for all outcomes (all *p* < 0.05), indicating that the combined treatment group showed greater improvements over time compared with the routine care group. The main effects of time and group were also significant for all outcomes (all *p* < 0.001). The group main effect was significant for HAMA, 6MWD, FVC, and FEV1 (all *p* < 0.01), but not for HAMD (*p* = 0.222, $\eta^{2}$ = 0.009).

**Table 6.** **Repeated-measures ANOVA results for all outcomes**.

Outcome	Effect	F (df)	*p*	$\eta^{2}$
HAMA	Time	128.5 (1, 166)	<0.001	0.436
	Time × Group	21.1 (1, 166)	<0.001	0.113
	Group	9.6 (1, 166)	0.002	0.055
HAMD	Time	94.2 (1, 166)	<0.001	0.362
	Time × Group	9.3 (1, 166)	0.040	0.025
	Group	1.5 (1, 166)	0.222	0.009
6MWD	Time	87.5 (1, 166)	<0.001	0.345
	Time × Group	20.3 (1, 166)	<0.001	0.109
	Group	14.2 (1, 166)	<0.001	0.079
FVC	Time	36.6 (1, 166)	<0.001	0.180
	Time × Group	5.2 (1, 166)	0.023	0.031
	Group	7.8 (1, 166)	0.006	0.045
FEV1	Time	71.2 (1, 166)	<0.001	0.300
	Time × Group	19.6 (1, 166)	<0.001	0.106
	Group	22.3 (1, 166)	<0.001	0.118

Note: df, degrees of freedom; $\eta^{2}$, partial eta squared; HAMA, Hamilton Anxiety Scale; HAMD, Hamilton Depression Scale; 6MWD, 6-minute walk distance; FVC, forced vital capacity; FEV1, forced expiratory volume in one second.

## Discussion

COPD is the second most common respiratory disease worldwide. A recent systematic review and meta analysis reported that the global prevalence of COPD among adults aged 40 years and older was 12.64% (95% CI 10.75%–14.65%) based on the fixed ratio spirometry criterion (FEV1/FVC < 0.70) [1]. Although asthma is more common, COPD is associated with higher morbidity and mortality. As a common, progressive respiratory disease, COPD is one of the leading causes of disability and death worldwide, causing long-term and profound negative impacts on patients’ physical and mental health and quality of life [17]. The incidence and prevalence of anxiety and depression are significantly higher in patients with COPD [18], with an estimated one-third of patients with COPD exhibiting depression related to symptom presentation. Study has shown that the prevalence of anxiety in patients with COPD ranges from 7% to 50%, and the prevalence of depression ranges from 10% to 57% [19]. Anxiety and depression can interfere with the management of COPD, forming a pathological cycle of deteriorating health status and reducing adherence to treatment and pulmonary rehabilitation [4]. This “mind–body” comorbidity substantially exacerbates the overall burden of the disease, ultimately leading to problems such as increased hospitalization risk, rising medical costs and severely impaired quality of life for patients [20]. Therefore, exploring a comprehensive treatment model that can improve both the physiological function and psychological state of patients with COPD has become a key issue to be addressed in the current COPD rehabilitation research.

Physical exercise is the cornerstone of COPD rehabilitation and one of the most effective non-pharmacological treatments. Unlike pharmacological treatment, which primarily relieves symptoms and targets acute exacerbations, exercise targets the systemic manifestations of COPD, including skeletal muscle dysfunction, reduced exercise capacity, and impaired quality of life [21]. A review suggests that structured exercise training, including aerobic exercise, strength training, and interval training, not only improves physical function but also reduces inflammation and oxidative stress, potentially slowing disease progression and lowering the risk of exacerbations [22]. Emerging transcriptomic evidence across diverse diseases, including respiratory disorders, has further highlighted the involvement of CD4+ T lymphocyte gene expression profiles in disease pathophysiology [23], which may offer a mechanistic basis for understanding the immunomodulatory effects of exercise-based rehabilitation. However, conventional rehabilitation programs often focus on restoring physical function with limited attention to the psychological and emotional problems commonly experienced by patients. The treatments are relatively simple, which may reduce patients’ adherence to the rehabilitation training due to emotional disorders [24]. At the same time, psychological treatments such as MBSR are increasingly applied in the management of chronic diseases. It is hypothesized that mindfulness-based treatments promote emotion regulation through non-judgmental present-moment awareness and a shift in stress perception, thereby improving cardiopulmonary health and ultimately reducing the stress response [25]. MBSR has been found to help reduce anxiety; a network meta analysis found that MBSR combined with Baduanjin significantly reduced anxiety and depression in patients with COPD compared with usual care [26]. However, while psychological treatment alone can improve mood, its direct impact on core physiological indicators such as lung function and exercise capacity is limited.

Based on this, the integration of exercise rehabilitation and MBSR and the IMB model as a theoretical framework are expected to promote the formation and maintenance of healthy behaviours and achieve synergistic improvement of mind and body through the reinforcement of information, motivation and behavioural skills.

Our study found that the combination of exercise rehabilitation and MBSR, implemented within the IMB framework, was associated with clinical improvements. The observed improvements may be explained by synergistic mechanisms: exercise provides behavioural activation while MBSR enhances emotional regulation. The within-group improvement in 6MWD for the combined treatment group (172.98 m) exceeded the minimal clinically important difference (MCID) of 54 m for COPD [27], and the within-group reduction in mMRC also surpassed the reported MCID of 1.0 point [28], indicating that the combined treatment produced meaningful improvements in exercise capacity and dyspnoea severity compared with baseline. However, the between group differences in changes did not reach the respective MCID thresholds, suggesting that the additional benefit of the combined treatment over routine care alone, although statistically significant, may be limited in clinical relevance. Although the IMB framework served as an organizational structure for delivering the combined program, the current study design does not allow us to isolate its independent contribution. Compared with prior studies examining exercise or MBSR alone, our findings suggest that combining both within the IMB framework may yield concurrent improvements in psychological and physical benefits.

Despite the positive results, this study has several limitations.

First, it was a retrospective, single-centre study, which may be subject to selection bias. Crucially, enrolment in the combined program was voluntary, introducing the potential for volunteer bias, as patients who chose to participate may have been more motivated, health-conscious, or had better social support than those who declined, potentially inflating the observed treatment effects. Because patients were not randomized and group assignment was based on documented care received, there is a high risk of confounding by indication and other unmeasured factors. Causality cannot be inferred.

Second, the control group received only routine care, whereas the experimental group received a multicomponent treatment (IMB-based education combined with exercise rehabilitation and MBSR). The absence of control arms receiving exercise alone or MBSR alone makes it impossible to determine whether the observed benefits are attributable to the combination of components, to the IMB framework specifically, or to nonspecific factors such as increased attention. Consequently, the current design does not allow the independent contribution of the IMB model to be isolated from those of exercise and MBSR.

Third, the sample size was limited and originated from a single organization, which resulted in some limitations on the generalizability of the study results.

Fourth, the 12-week program, although covering an important stage of behavioural development, could not assess the long-term sustainability of the effects of this integrated treatment model.

Finally, the study relied on self-reported scales (HAMA, HAMD) as primary outcome measures. Although these scales have good reliability and validity, the absence of objective physiological indicators (e.g., inflammatory markers or cortisol levels) and third-party assessments may have introduced measurement bias.

Based on the results of this study and existing research progress, future research can be further explored in the following directions. First, in terms of study design, multiple control groups (e.g., exercise rehabilitation group alone, MBSR group alone) can be set up to clarify the independent effects of exercise rehabilitation and MBSR as well as their interactions, so as to optimize the composition of the treatment protocols and achieve the precise allocation of treatment resources. Second, large-scale, multicentre, randomized controlled trials are needed to recruit patients from different regions and medical institutions, covering a wider range of ages, disease durations, and pulmonary function stages, to improve the external validity and generalizability of the research results. Third, it is recommended to combine biomarkers (e.g., inflammatory factors, cortisol, and neurotransmitters) and neuroimaging techniques to explore in depth the physiological-psychological mechanisms of the IMB model in modulating the interaction between exercise rehabilitation and MBSR, providing a more solid theoretical basis for optimizing treatment programs. Fourth, the follow-up period should be extended to assess the impact of the treatment on patients’ long-term quality of life, utilization of medical resources, and socioeconomic burden. Furthermore, future research could explore combining IMB model-based treatments with digital tools (e.g., mobile applications and wearable devices) to improve patient adherence and the sustainability of self-management, potentially overcoming geographical and time limitations. In addition, qualitative research is encouraged to gain a deeper understanding of patients’ acceptance and experience with the treatment program, providing a reference for its promotion in clinical practice.

## Conclusions

This retrospective study observed an association between the combination of exercise rehabilitation and MBSR, delivered within the IMB framework, and improvements in psychological status, dyspnoea, exercise tolerance, and lung function in stable patients with COPD and anxiety and depression. However, because the IMB framework was not tested independently (e.g., via a control arm receiving exercise plus MBSR without the IMB framework), these associations cannot be attributed specifically to the IMB model. Future large-scale, multicentre, randomized controlled trials with factorial designs are needed to confirm these findings and to clarify the specific role of the IMB model in comprehensive COPD rehabilitation.

## Availability of Data and Materials

All data included in this study can be obtained by contacting the corresponding author if needed.

## References

[1] Al Wachami N, Guennouni M, Iderdar Y, Boumendil K, Arraji M, Mourajid Y (2024). Estimating the global prevalence of chronic obstructive pulmonary disease (COPD): a systematic review and meta-analysis. *BMC Public Health*.

[2] Pascoe A, Chen X, Smallwood N (2025). A narrative review of proactive palliative care models for people with COPD. *Therapeutic advances in respiratory disease*.

[3] Siraj RA (2025). COPD and Comorbid Mental Health: Addressing Anxiety, and Depression, and Their Clinical Management. *Medicina (Kaunas, Lithuania)*.

[4] Volpato E, Toniolo S, Pagnini F, Banfi P (2021). The Relationship Between Anxiety, Depression and Treatment Adherence in Chronic Obstructive Pulmonary Disease: A Systematic Review. *International journal of chronic obstructive pulmonary disease*.

[5] Lamberton CE, Mosher CL (2024). Review of the Evidence for Pulmonary Rehabilitation in COPD: Clinical Benefits and Cost-Effectiveness. *Respiratory care*.

[6] Zhang M, Khan MU (2026). The Effects of Aerobic Exercise on Prognosis, Quality of Life, and Psychological Outcomes of Patients With Chronic Obstructive Pulmonary Disease: A Systematic Review and Meta-Analysis. *Chronic obstructive pulmonary diseases (Miami, Fla.)*.

[7] Fortes PP, Dos Santos-Ribeiro S, de Salles-Andrade JB, Moreira-de-Oliveira ME, de Abreu-Cervone F, de Faro LFT (2025). Mindfulness interventions and quality of life in anxiety-related disorders: A systematic review and meta-analysis. *Journal of affective disorders*.

[8] Liang Y, Cao S, Xu H, Wang S, Feng M, Wang J (2024). Apply the information-motivation-behavioral model to explore the relationship between oral health literacy and oral health behaviors among community-dwelling older adults. *BMC Public Health*.

[9] Agustí A, Celli BR, Criner GJ, Halpin D, Anzueto A, Barnes P (2023). Global Initiative for Chronic Obstructive Lung Disease 2023 Report: GOLD Executive Summary. *The European respiratory journal*.

[10] Shi X, Shi L, Tian Y, Li N (2025). Comparative efficacy of abdominal tuina and mosapride citrate in functional constipation: A clinical trial. *Medicine*.

[11] Lei X, Ji W, Guo J, Wu X, Wang H, Zhu L (2022). Research on the Method of Depression Detection by Single-Channel Electroencephalography Sensor. *Frontiers in psychology*.

[12] Xiong T, Bai X, Wei X, Wang L, Li F, Shi H (2023). Exercise Rehabilitation and Chronic Respiratory Diseases: Effects, Mechanisms, and Therapeutic Benefits. *International journal of chronic obstructive pulmonary disease*.

[13] Hoge EA, Bui E, Mete M, Dutton MA, Baker AW, Simon NM (2023). Mindfulness-Based Stress Reduction vs Escitalopram for the Treatment of Adults With Anxiety Disorders: A Randomized Clinical Trial. *JAMA Psychiatry*.

[14] Zandifar A, Panahi M, Badrfam R, Qorbani M (2024). Efficacy of empagliflozin as adjunctive therapy to citalopram in major depressive disorder: a randomized double-blind, placebo-controlled clinical trial. *BMC Psychiatry*.

[15] Ribeiro S, Cardoso CS, Valério M, Machado J, Costa J, Rodrigues C (2022). Confirmatory Evaluation of the Modified Medical Research Council Questionnaire for Assessment of Dyspnea in Patients with Chronic Obstructive Pulmonary Disease in Portugal. *Acta médica portuguesa*.

[16] Ferté JB, Boyer FC, Taiar R, Pineau C, Barbe C, Rapin A (2022). Impact of resistance training on the 6-minute walk test in individuals with chronic obstructive pulmonary disease: A systematic review and meta-analysis. *Annals of physical and rehabilitation medicine*.

[17] Halpin DMG, Criner GJ, Papi A, Singh D, Anzueto A, Martinez FJ (2021). Global Initiative for the Diagnosis, Management, and Prevention of Chronic Obstructive Lung Disease. The 2020 GOLD Science Committee Report on COVID-19 and Chronic Obstructive Pulmonary Disease. *American journal of respiratory and critical care medicine*.

[18] Hurtado-Ruzza R, Álvarez-Calderón-Iglesias Ó, Becerro-de-Bengoa-Vallejo R, Calvo-Lobo C, San-Antolín M, Losa-Iglesias ME (2022). Self-reported depression and anxiety among COPD patients. A case-control study. *São Paulo medical journal = Revista paulista de medicina*.

[19] Yohannes AM, Casaburi R, Dryden S, Hanania NA (2022). The effectiveness of pulmonary rehabilitation on chronic obstructive pulmonary disease patients with concurrent presence of comorbid depression and anxiety. *Respiratory medicine*.

[20] Zhao X, Liu G, Liu D, Zou L, Huang Q, Chen M (2024). Clinical and economic burden of anxiety/depression among older adult COPD patients: evidence from the COPD-AD China Registry study. *Frontiers in psychiatry*.

[21] Troosters T, Janssens W, Demeyer H, Rabinovich RA (2023). Pulmonary rehabilitation and physical interventions. *European respiratory review: an official journal of the European Respiratory Society*.

[22] Liu S, Yang A, Yu Y, Xu B, Yu G, Wang H (2025). Exercise Prescription Training in Chronic Obstructive Pulmonary Disease: Benefits and Mechanisms. *International journal of chronic obstructive pulmonary disease*.

[23] Liao H, Ma Q, Chen L, Guo W, Feng K, Bao Y (2025). Machine learning analysis of CD4+ T cell gene expression in diverse diseases: insights from cancer, metabolic, respiratory, and digestive disorders. *Cancer genetics*.

[24] Qin H, Jia P, Yan Q, Li X, Zhang Y, Jiang H (2025). Barriers and facilitators to pulmonary rehabilitation in COPD: a mixed-methods systematic review. *BMC pulmonary medicine*.

[25] Jeong J, Hu Y, Zanuzzi M, DaCosta D, Sabino-Carvalho JL, Li S (2025). Autonomic modulation with mindfulness-based stress reduction in chronic kidney disease: a randomized controlled trial. *The Journal of physiology*.

[26] Tang Y, Wang J, Jiang Y (2026). Comparative effects of mind-body exercises on anxiety and depression in patients with chronic obstructive pulmonary disease: A network meta-analysis of randomized controlled trials. *Heart & lung: the journal of critical care*.

[27] Redelmeier DA, Bayoumi AM, Goldstein RS, Guyatt GH (1997). Interpreting small differences in functional status: the Six Minute Walk test in chronic lung disease patients. *American journal of respiratory and critical care medicine*.

[28] Cheng SWM, McKeough ZJ, McNamara RJ, Alison JA (2023). Pulmonary Rehabilitation Using Minimal Equipment for People With Chronic Obstructive Pulmonary Disease: A Systematic Review and Meta-Analysis. *Physical therapy*.

